# Supplementary material for: Characterizing the flavodoxin landscape in Clostridioides difficile
Source: Microbiol Spectr. 2024 Feb 6;12(3):e01895-23. doi: 10.1128/spectrum.01895-23 (PMC10913485; doi:10.1128/spectrum.01895-23)
Supplement: Fig. S1 to S4 — Supplementary figures. [file spectrum.01895-23-s0001.pdf]

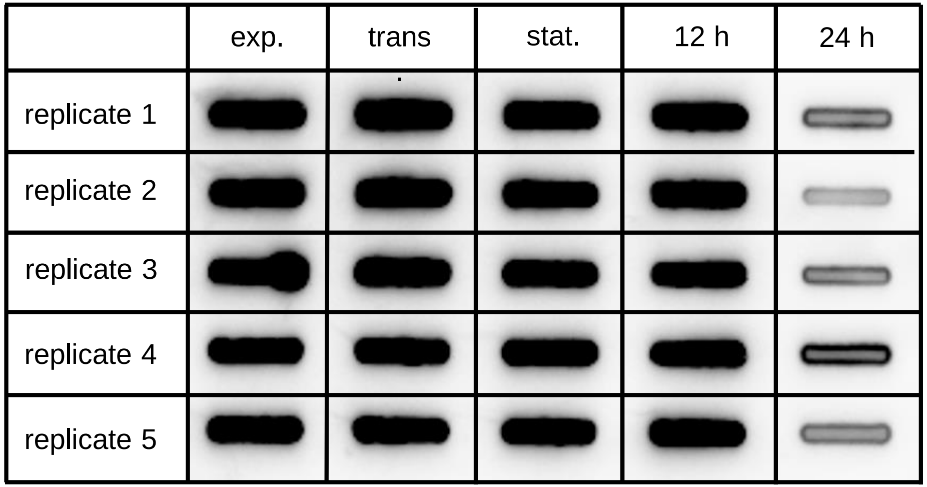

FIG S1: Expression profile of 5S rRNA from *C. difficile* 630 during growth. Image of 5 µg RNA slot blot analyses hybridized with the 5S rRNA probe (1:10.000 diluted) in five biological replicates. The detection time was 1 min.

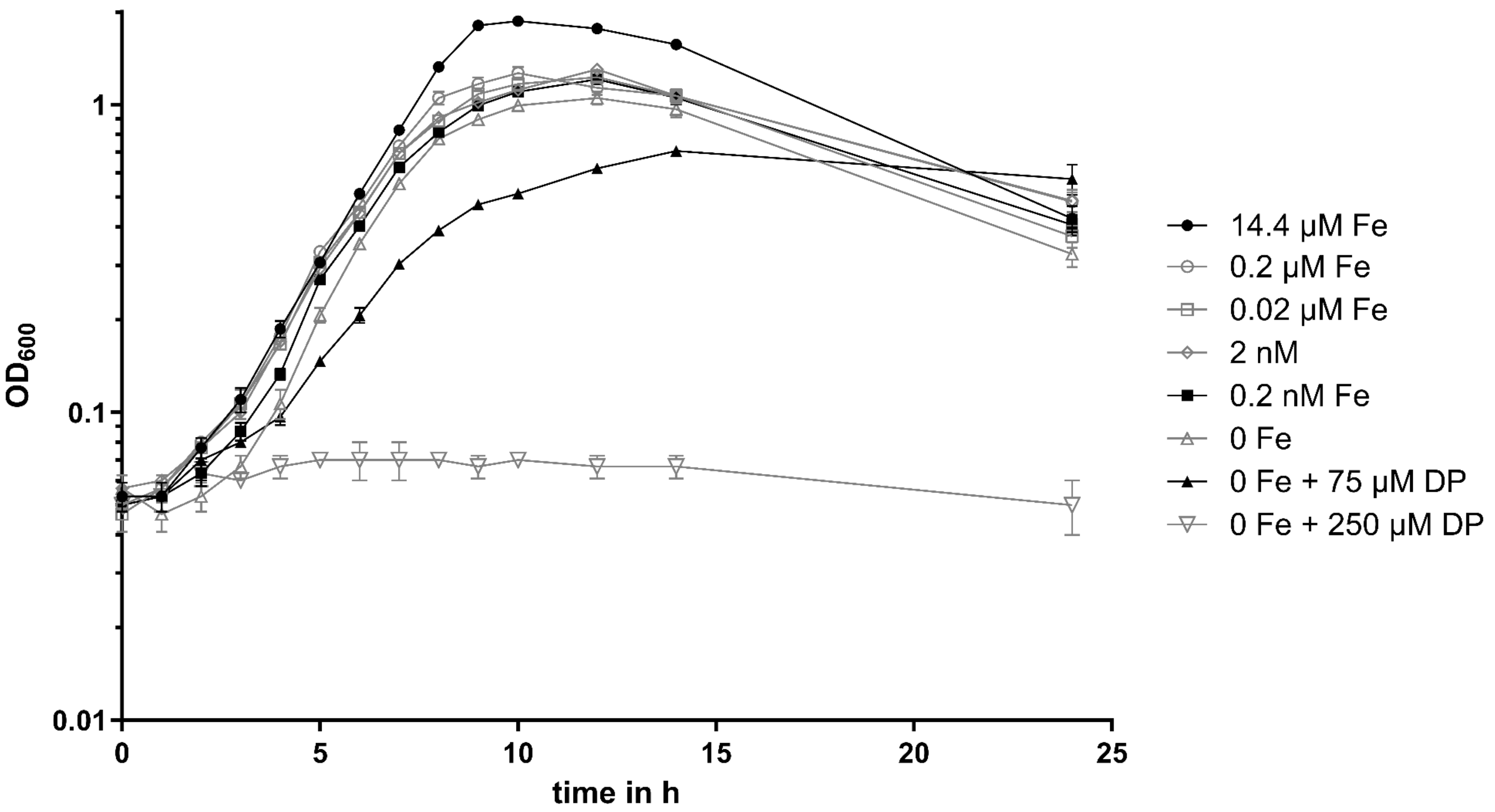

FIG S2: Growth curves of *C. difficile* 630 in CDMM with different concentrations of iron. *C. difficile* 630 was cultivated in CDMM supplemented with a total iron concentration of 14.4 µM, 0.2 µM, 0.02 µM, 2 nM, 0.2 nM, 0 Fe, 0 Fe with 75 µM DP or 0 Fe with 250 µM DP, respectively. Growth was monitored in three independent cultivations. Standard deviations are indicated. Fe - iron, DP - 2,2‘-dipyridyl

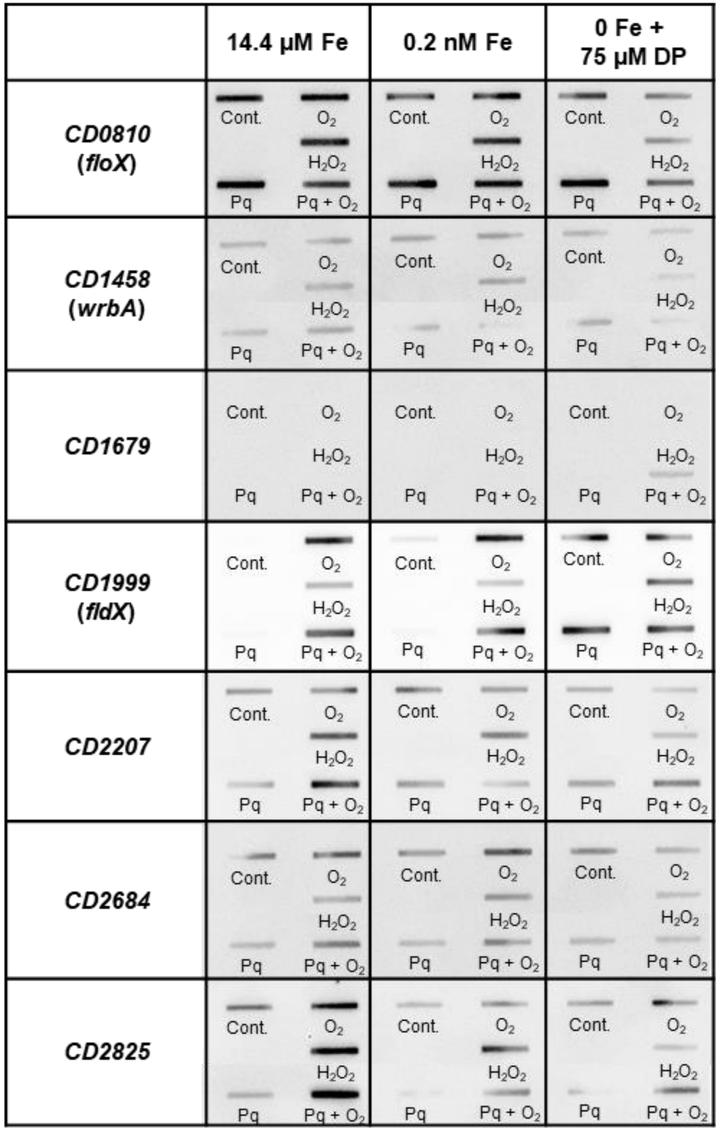

FIG S3: Flavodoxin expression analyses under different oxidative stress conditions and iron limitation. Image of different slot blots, hybridized with the probes named on the left. Out of three replicates per blot one representative blot for each flavodoxin is shown. The detection time was 1 min for each blot.

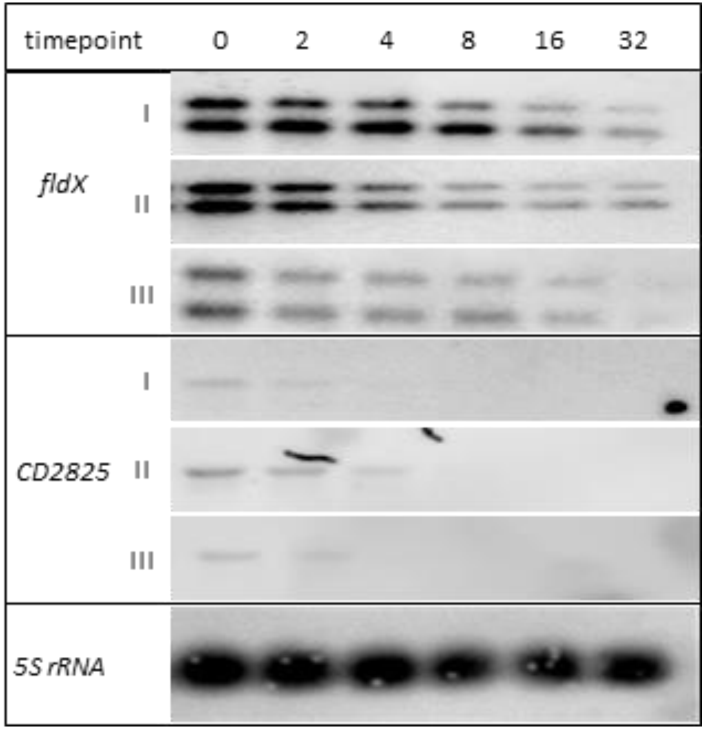

FIG S4: Northern blot images of fldX, CD2825 and 5S rRNA for the determination of transcript half-lives. The half-lives of fldX and CD2825 were determined after 10 min of H2O2 stress. The signal from a 5S rRNA-specific probe served as a loading control. Images show three biological replicates (I, II, III). Harvesting timepoints are shown in minutes.
